# Biomaterial-Derived Calcium Carbonate Nanoparticles for Enteric Drug Delivery

**DOI:** 10.1155/2016/3170248

**Published:** 2016

**Authors:** Diane Render, Temesgen Samuel, Howard King, Madan Vig, Shaik Jeelani, Ramapuram Jayachandra Babu, Vijaya Rangari

**Affiliations:** 1 Department Materials Science and Engineering, Tuskegee University, Tuskegee, AL 36088, USA; 2 Department of Pathobiology, Tuskegee University, Tuskegee, AL 36088, USA; 3 Department of Clinical Sciences, Tuskegee University, Tuskegee, AL 36088, USA; 4 Department of Drug Discovery and Development, Harrison School of Pharmacy, Auburn University, Auburn, AL 36849, USA

## Abstract

Oral drug delivery systems provide the most convenient, noninvasive, readily acceptable alternatives to parenteral systems. In the current work, eggshell-derived calcium carbonate (CaCO_3_) nanoparticles were used to develop enteric drug delivery system in the form of tablets. CaCO_3_ nanoparticles were manufactured using top-down ball-milling method and characterized by X-ray diffractometry (XRD) and transmission electron microscopy (TEM) and loaded with 5-fluorouracil as a model drug. Tablets with varying CaCO_3_ core and binder compositions were fabricated and coated with Eudragit S100 or Eudragit L100. Suitability for enteric delivery of the tablets was tested by oral administration to rabbits and radiography. Radiograph images showed that the tablet remained in the stomach of the rabbit for up to 3 hours. Further modifications of these biomaterial-derived nanoparticles and the coatings will enable manufacturing of stable formulations for slow or controlled release of pharmaceuticals for enteric delivery.

## 1. Introduction

Calcium carbonate (CaCO_3_) is the most abundant mineral in nature, which makes it a cheap, inorganic material [1]. Synthetic forms of CaCO_3_ have been used in oil, paint, paper, plastics, coatings, environmentally friendly items, calcium-enriched foods, drug delivery, templates for microcapsules, and bone filling material [2–4].

Drug administration by enteric route is a much desired delivery system because it offers the most convenient, readily acceptable, and noninvasive alternative. Despite these advantages, enteric delivery of drugs is also beset by variant microenvironment in the digestive system, including pH, enzymatic, mucus, and microbial barriers [5]. Therefore, different approaches that address the barriers above are being sought [6, 7].

Enterically delivered drugs could have systemic or local effects in the digestive tract. The colon is one of the organ sites for which enteric delivery of drugs has become a subject of research [8]. Such systems are designed to respond to pH, enzymes, and microbial flora and for both local and systemic activities. Colonic drug delivery has become increasingly desirable because of the benefits of a sufficient amount of treatment at the diseased site. Colonic drug delivery systems are created in order to increase the efficacy and reduce the side effects of drugs delivered to the site [9]. In order to successfully deliver drugs to the colonic region, they have to pass the stomach and small intestine and then be released at the colon site [10]. For example, colon-targeted delivery of cytotoxic drugs such as 5-fluorouracil (5-FU), commonly used to treat colon cancer [11, 12], may reduce the side effects following systemic delivery while achieving high local concentrations.

In this study, we evaluated the suitability of calcium carbonate (CaCO_3_) nanoparticle-based tablets for enteric drug delivery systems. We used eggshells as raw material for the synthesis of nanoparticles. Eggshells have organic and inorganic components, which can be used for different applications. The outside layers of the eggshells are composed of 95% CaCO_3_ and the rest 5% include calcium phosphate, magnesium carbonate, and soluble and insoluble proteins, yielding the primary benefit of using a biological source of CaCO_3_. Eggshells were chosen for this research because they are highly porous, low-cost, easily accessible, recyclable, and biocompatible with the human body.

The tablets were coated with Eudragit S100 or Eudragit L100, a pH-sensitive methacrylic acid-methyl methacrylate copolymer [13], to prevent disintegration of tablets in the stomach. Tablet gastric transit dynamics after oral administration in rabbits were tracked by radiography.

## 2. Materials and Methods

### 2.1. Materials

Eggshells were obtained from American Dehydrated Foods Inc. (Social Circle, GA). Polypropylene glycol (PPG), ethanol, and glycerol were obtained from Sigma Aldrich (St. Louis, MO). Hydroxypropyl methylcellulose (HPMC E4M) and starch were obtained as gift samples from Colorcon (West Point, PA). Silicified microcrystalline cellulose (SMCC) was obtained from Penwest Pharmaceuticals (Patterson, NY).

### 2.2. Preparation of CaCO_3_ Nanoparticles

Following initial cleaning, mechanochemical milling and sonochemical irradiation were carried out in the synthesis of CaCO_3_ nanoparticles. The eggshells were boiled for 24 hours to deactivate and remove proteins. After the eggshells were cleaned, they were placed into a blender for initial grinding. The ground eggshells were dispersed in a water bath for 24 hours and finally washed with fresh water and dried in an oven at 50°C. After the particles were dried, 1000 mg of the particles were placed in a ball-mill canister with 5mL of PPG and ball-milled for 10 hours. After the eggshells were ball-milled, they were washed three times with ethanol and dried for 24 hours in a vacuum oven at room temperature. After the particles were dried, they were sifted through a 20 *μ*m pore sifter to remove aggregates and to obtain uniform size particles. These particles were further irradiated with high intensity ultrasonic horn to reduce the particle sizes to nanoscale for three hours in 50 mL of glycerol. The particles were then washed three more times and dried for 24 hours in a vacuum drier and later dried again in a vacuum oven at 100°C for 24 hours.

### 2.3. Characterization

X-ray diffraction (XRD) was used to determine the structure and purity of the synthesized CaCO_3_ nanoparticles in comparison with calcite JCPDS data file. The material was tested on a Rigaku D/MAX 2200 X-ray diffractometer. A powdered sample was placed on a sample holder and tested for XRD studies. Transmission electron microscope (TEM) observation was done using a Hitachi H7600 microscope. TEM analysis was conducted to identify the morphology and size of the CaCO_3_ nanoparticles. The nanoparticles were dispersed in ethanol and then placed onto a copper sample grid by drops of the solution and then air-dried.

### 2.4. Drug Loading Studies on CaCO_3_ Nanoparticles

The target for drug loading concentration in this study was ~100 *μ*M for each batch of CaCO_3_ nanoparticles. A stock solution of 5-FU with a concentration of 100 mM has been created with deionized water. 900 *μ*L of dH_2_O was combined with 100 *μ*L of 5-FU from the stock solution. The samples were processed until the particles were saturated for 24 hours. The samples were frozen down in a freezer (−20°C); then, caps were removed and replaced with Parafilm^™^ with small holes poked so that the samples could sublime while freeze-drying. The samples were freeze-dried in a Modulyo Freeze-dryer by Thermo Fisher Scientific (Hudson, New Hampshire) for 24 hours. The nanoparticles were tested for the 5-FU content assay using high performance chromatography (HPLC).

### 2.5. Preparation of Tablets

All the formulations (F1–F5) contained the nanoparticles and excipients in the similar ratio, but the tablets weight was varied between 90 and 500 mg (Table 1). The different tablet weights were created to determine the one that is suitable for easy administration in rabbits and that can traverse stomach and duodenum without any difficulty as tracked by the radiography studies. The tablet formulation consisted of CaCO_3_ nanoparticles, starch, hydroxypropyl methylcellulose (HPMC E4M), and silicified microcrystalline cellulose (SMCC). The tablet blend contained 1 mg of 5-FU per 100 mg of CaCO_3_ nanoparticles and 5-FU content was varied based on the tablet weight as shown in Table 1. The tablets were compressed on a hydraulic Carver press model-B (Carver Inc., Wabash, IN) at a compression force of 3000 pounds per square inch. The tablet formulation F2 was subjected to enteric coating to achieve delayed release of 5-FU in the intestinal region. An aqueous 30% latex polymer solution of Eudragit S100 or Eudragit L100 was used as a coating polymer. Fifteen coats were sequentially layered using a spray gun to each tablet to achieve a coating thickness of ~120 microns [14]. The cross section of the tablet was imaged by a scanning electron microscope (SEM; JEOL-JSM-5800, Tokyo, Japan) to show the tablet core and coat thickness. The micrographs were obtained at an excitation voltage of 12 kV and magnification factors of ×1,000. Hardness of the tablets was determined using digital hardness tester. Friability of the tablets was determined by model F2 Tablet Friabulator. Disintegration time of the tablets was determined by model D2 disintegration tester (Pharma Alliance Group, Valencia, CA).

### 2.6. Dissolution Studies of Tablets

For dissolution studies, solutions of 0.1N hydrochloric acid (HCl) (pH 2.0) and phosphate buffered saline (PBS) (pH 7.4) were prepared. The pH 2.0 was chosen to mimic the acidity levels of the stomach and pH 7.4 was chosen to mimic the basic levels of the small intestine and large intestine. The coated tablets were placed in 50 mL of HCl for two hours and 1.0 mL samples were taken and replaced every hour. Immediately after two hours, the tablets were transferred into 50 mL of PBS and 1.0 mL samples were taken and replaced at 0.5, 1, 2, 3, 4, and 5 hours. The samples obtained from the study using PBS were assayed using HPLC at 265 nm.

### 2.7. High Performance Liquid Chromatography

A HPLC system (Waters Corporation, Massachusetts, USA) equipped with 717 plus autosampler, 1525 pump, 2998 PDA UV detector, and Empower 2 software was used. Samples were eluted on a 4.6mm× 250mm Luna C18 analytical column with 5 *μ*m silica particles of 100 Å pore size (Phenomenex, Torrance, California, USA). The mobile phase consisted of 98% water and 2% methanol, which was run at a flow rate of 1.0 mL/min.

### 2.8. Gastric Transit Studies in Rabbits

For *in vivo* testing, New Zealand White Rabbits were used. Procedures for *in vivo* studies were structured to mimic the pathway a tablet would follow if orally administered. The rabbits were held in a “scruffing” position, where the back is fully supported. A tablet popper with a plunger was used to slide the tablet behind the tongue base of manually restrained rabbits. The animals were allowed to swallow the tablets and were observed for 1–2 minutes to ensure swallowing. Radiographs were taken using a small animal radiography unit (Tuskegee University Small Animal Clinic, Tuskegee, AL) before, immediately after, and every hour after the tablet administration. These *in vivo* studies were conducted after approval by the Tuskegee University Animal Care and Use Committee.

## 3. Results and Discussion

### 3.1. Characterization

**Figure 1:**
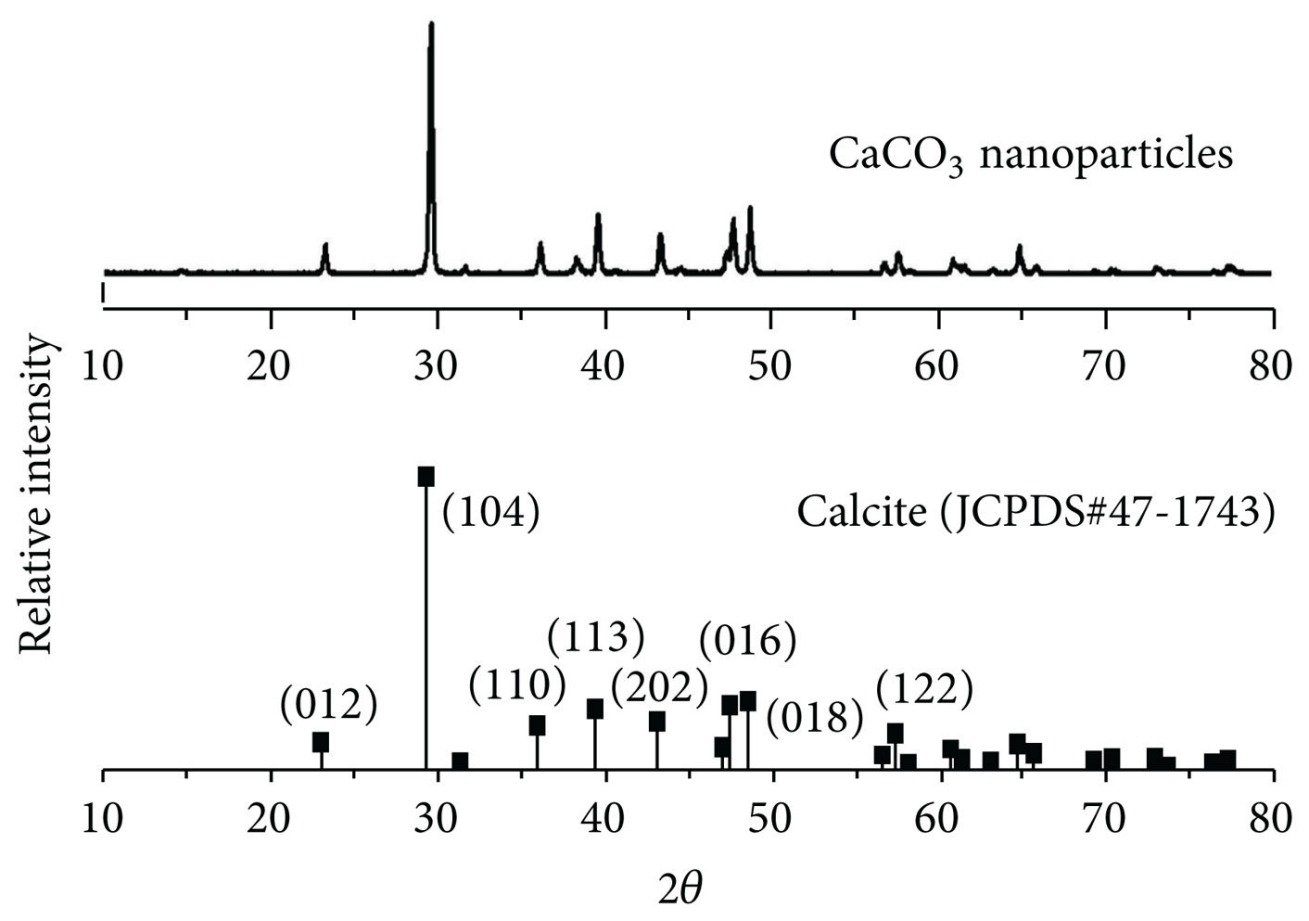
X-ray diffraction spectra of CaCO_3_ nanoparticles and JCPDS standard.

The XRD patterns were studied for calcium carbonate nanoparticles. Figure 1 shows the XRD spectrum of nanoparticles. These results show that the particles are highly crystalline and all the peaks match very well with the diffraction peaks of calcite CaCO_3_ (JCPDS card number 47-1743). No other impurities were observed.

### 3.2. Transmission Electron Micrograph and Size Distribution of CaCO_3_ Nanoparticles

**Figure 2:**
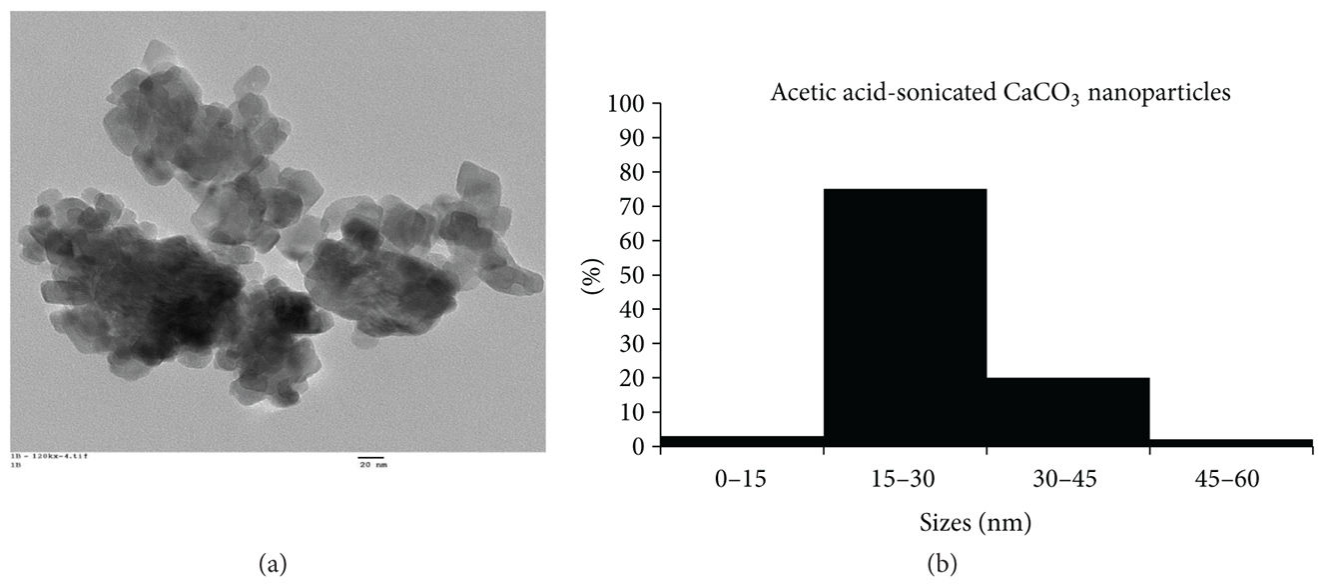
(a) Transmission electron micrograph and (b) size distribution chart of CaCO_3_ nanoparticles.

Figure 2(a) shows a transmission electron micrograph of CaCO_3_ nanoparticles and Figure 2(b) shows a size distribution chart accumulated from several transmission electron micrographs of the nanoparticles. The micrograph verifies the size and morphology of the nanoparticles and the particle dimensions were also used to create a size distribution chart. These nanoparticles possess sizes ranging from ~10 to 60 nm, with 75% of their uniform distribution in the 15–30 nm range. The nanoparticles outside of 15–30 nm range are attributed to their agglomeration while being in preparation for TEM.

### 3.3. Drug Loading of Nanoparticles and Formulation of Tablets

The CaCO_3_ nanoparticles were loaded with 1 mg of 5-FU per 100 mg of the nanoparticles. The drug content of the loaded nanoparticles was determined by HPLC. As 5-FU was highly water soluble, we did not attempt to wash the nanoparticles following the drug loading study.

**Figure 3:**
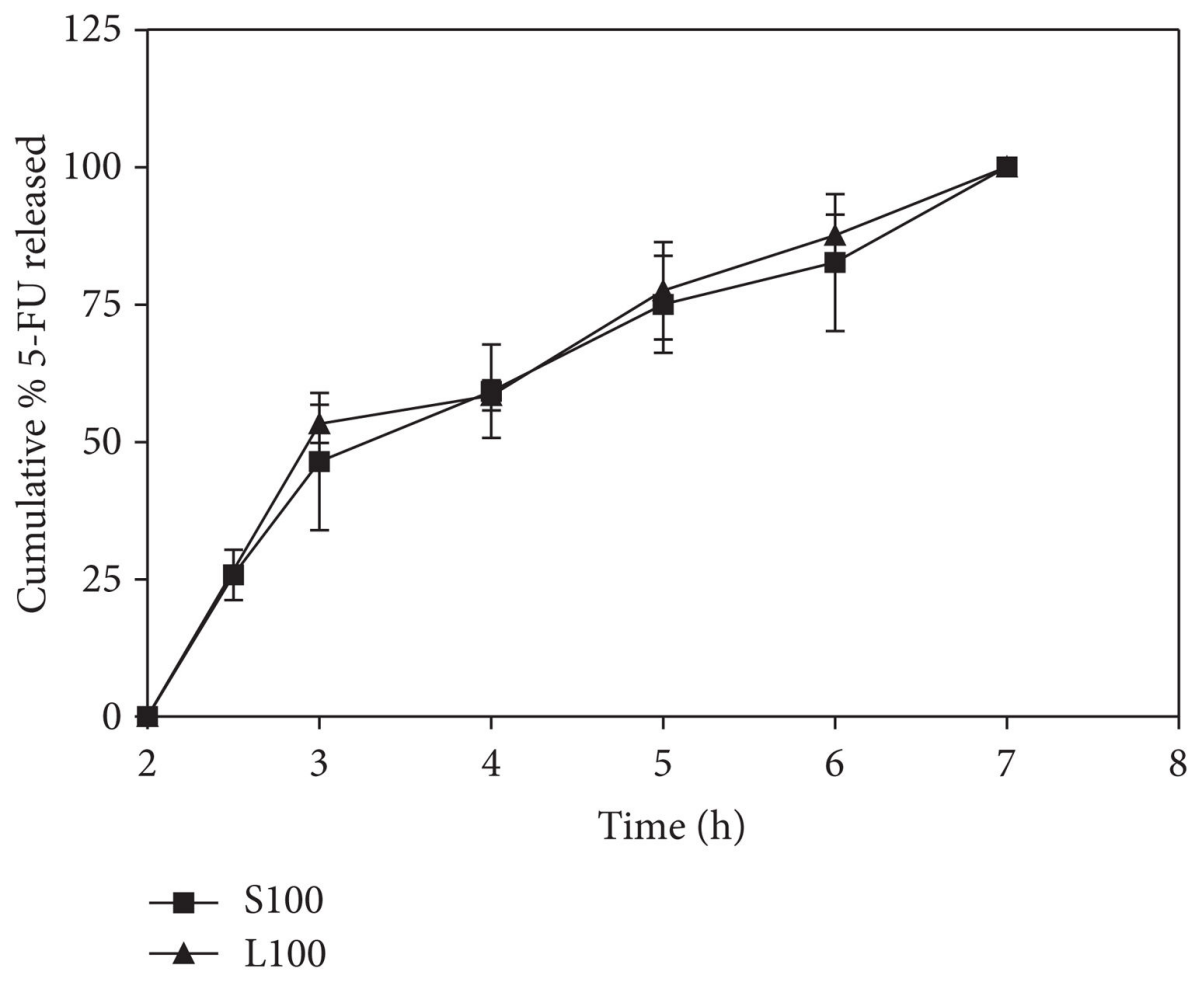
Dissolution of 5-FU from the coated tablets (F2).

We made use of the radiopaque properties of compacted CaCO_3_ nanoparticles for gastric transit studies. The fabricated tablets (Table 1) needed to contain sufficient amounts of CaCO_3_ to appear radiopaque on X-ray images and also accommodate the other components without compromising physical integrity and cohesiveness [15]. All the formulations (F1–F5) contained the nanoparticles and excipients in the similar ratio, but the tablets weight was varied in each case (90 mg–500 mg). Preliminary studies in rabbits indicated that formulation F2 was optimum for better retention in the stomach and transit to the intestine. The F2 tablets showed hardness of 12.6 ± 1.6 kp; friability of <0.1%, and the disintegration time of 30 min (in distilled water). As shown in the dissolution data (Figure 3), both Eudragit S100 and Eudragit L100 coatings could withstand the acid medium for 2 h and the tablets remained intact. Upon transferring the tablets to PBS pH 7.4 buffer, approximately half (Q 50%) of 5-FU was released in 3.5h and the entire drug (Q 100%) was released in about 6.5 hours.

**Figure 4:**
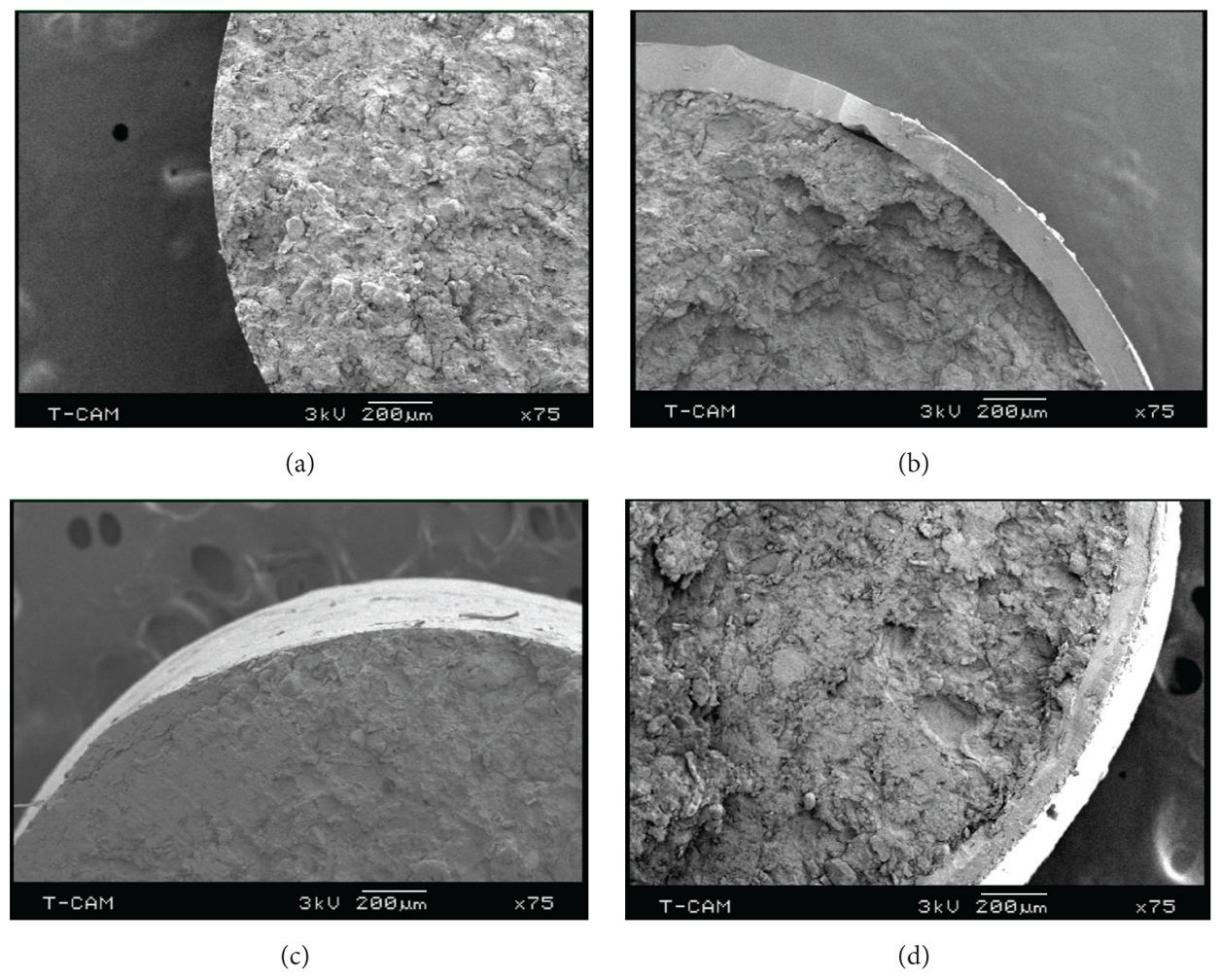
Scanning electron microscope images of noncoated and coated tablets (F2) to show the coat thickness is around 150 *μ*m.

Signs of a layer separation in formulation F2 suggested weakened bonding of the tablet constituents probably due to increased proportion of the CaCO_3_ at the expense of cohesion. Therefore, to minimize the possibility of physical defects arising before administration to rabbits, tablets were fabricated with reduced tablet weight and dimensions. The cross section of the tablet shows the coating thickness to be around 150 microns as determined by SEM(Figure 4). Tablets of formulation F5, which had the least total content (Table 1), were not detectable by radiography and, therefore, not further tested. After comparing tablet size, relative composition, the likelihood of easy oral administration, and gastric transit tests in rabbits, formulation F4 was chosen for further experiments.

### 3.4. Tablet Gastric Transit Studies

**Figure 5:**
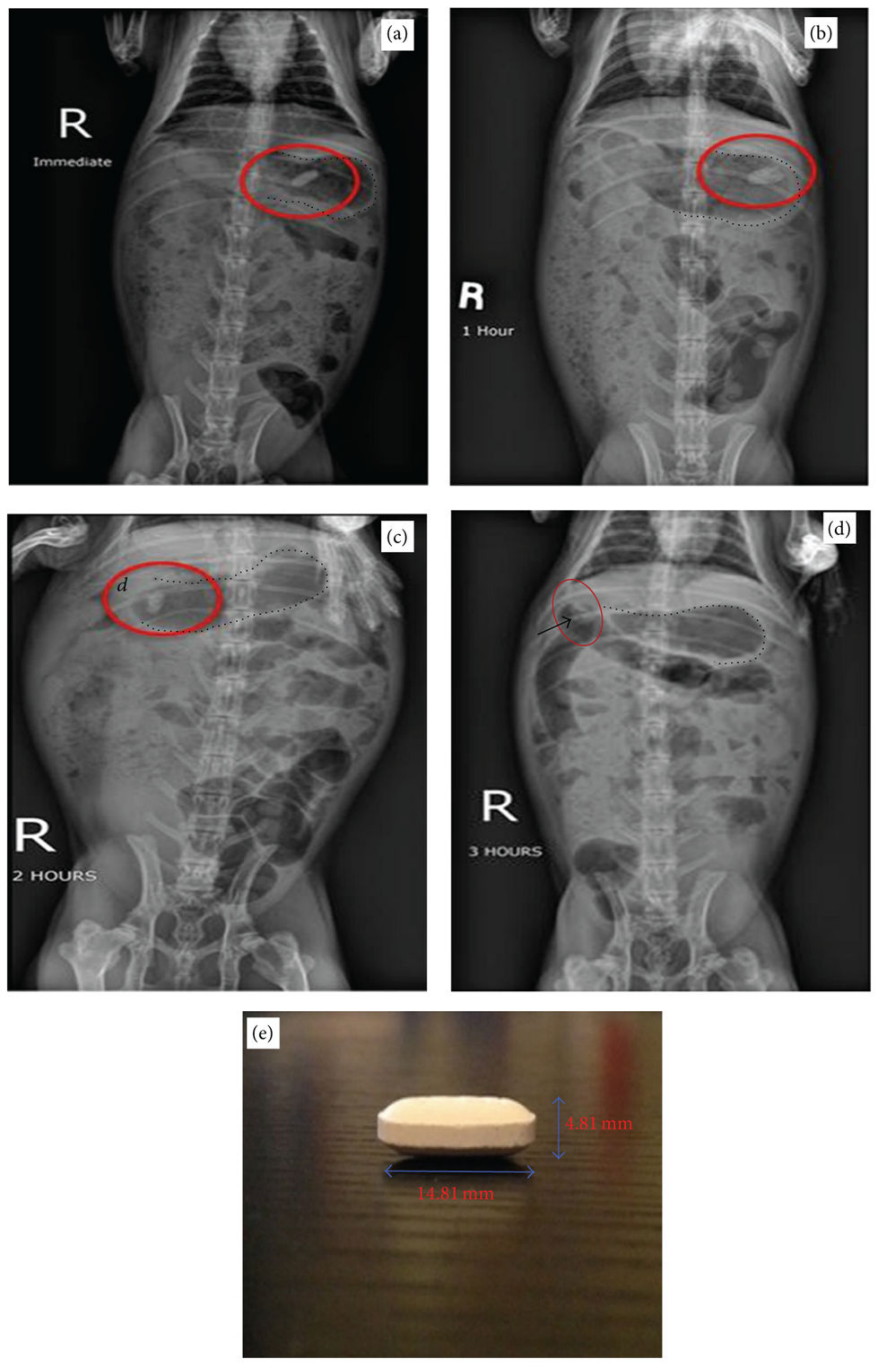
Gastric transit studies in rabbits using tablet formulation F2. ((a)–(d)) Radiographs taken immediately, 1 hour, 2 hours, and 3 hours after oral administration, respectively. (e) Dimensions of the tablet (length = 14.81 mm, height = 4.81 mm). Red ellipses and arrow in (d) show positions of the tablet, and dashed lines outline the rabbit stomach. *d*: location of the rabbit duodenum.

Tablets fabricated based on F1 could not hold the entire 500 mg content together and therefore were abandoned. Therefore, we tested tablets based on formulation F2, which has the next highest content. A tablet according to formulation F2 held together well despite some minor signs of layer separation or capping. When administered to rabbits, this formulation was radiopaque enough and also maintained integrity for up to 3 hours (Figures 5(a)–5(d)) though some swelling was evident. At 3 hours mark, the tablet was detectable in the duodenum of the rabbit. However, the relative size and content of tablets of formulation F2 made them difficult to administer and also resulted in cracking as seen in Figure 5(b). Although tablets of formulation F3 contained 50% of formulation F1, the relative dimension and thickness of the tablets were considered nonconducive for easy administration to rabbits.

**Figure 6:**
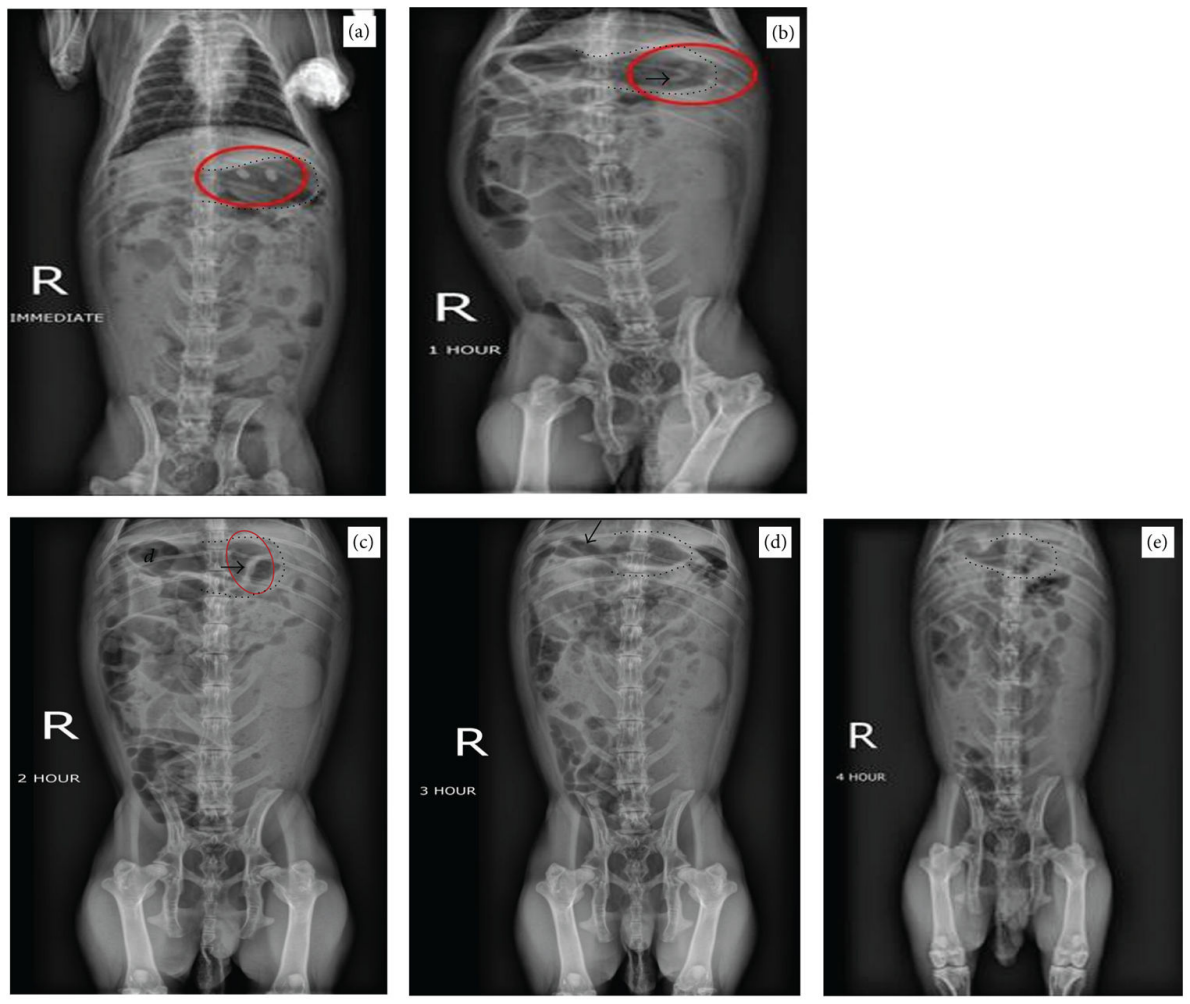
Gastric transit study in rabbits using tablets based on formulation F4 (a) immediately, (b) 1 hour, (c) 2 hours, (d) 3 hours, and (e) 4 hours after oral administration. Red ellipses in (a), (b), and (c) and arrows in (b), (c), and (d) show positions of the tablet, and dashed lines outline the rabbit stomach. *d*: location of the rabbit duodenum.

Next, gastric transit studies were conducted in rabbits using tablets based on formulation F4. Two tablets were administered. Figures 6(a) and 6(b) show both of the tablets in the stomach, immediately and 1 hour after administration. Figure 6(c) shows the tablets still in the stomach at 2 hours mark, which is visible by the increased opaqueness. Figure 6(d) shows that at 3 hours mark the tablets had lost integrity and only a faint radiopaque remnant of them was visible. Figure 6(e) shows that no tablet was detectable four hours after administration.

Overall, we found that tablet based on formulation F2 maintained its transit integrity slightly better than formulation F4 as it only slowly reduced in size during the gastric transit, despite signs of cracking. In either case, the time for detectable gastric transit for the tested tablets *in vivo* remained about 3 hours. At 4 hours mark, both formulations were undetectable. This duration for detectable transit in this study is the time it took from administration till the time the tablets were no longer detectable on radiographs. It is not clear how far beyond the stomach the tablets could move after they exit the stomach. These results from this gastric transit study show that stability in the stomach environment has been achieved. For ultimate drug delivery in the distal segments of the intestine, including the colon, such tablets need to traverse the GI tract with minimal loss of core integrity. Therefore, multiple layers of different chemistry may be deployed to enable delivery at the desired segment of the GI tract. Further studies are needed to modify these nanoparticles and tablet formulations for improved enteric drug delivery.

## 4. Conclusions

We have formulated tablets with potential for use in enteric drug delivery. The core component of the tablets is made of CaCO_3_ nanoparticles derived from eggshells. As such, this work also shows the salvage of waste-bound biomaterials for biomedical applications. Unlike chemically synthesized CaCO_3_ nanoparticles, eggshell-derived nanoparticles are porous, enabling drug loading. Additionally, nontoxic, biocompatible properties of these materials make them ideal candidates for biomedical applications [16]. By testing different tablet formulations, we were also able to identify optimal compositions of the tablets for gastric transit studies. A cylindrical tablet provided the best radiographs in the rabbit model and proved to have an easier passage through the upper digestive tract. Further studies that build upon these properties of CaCO_3_ tablets and nanoparticles, including multilayered formulations, will identify specific modifications that will enable distal enteric delivery of drug cargoes.

## Figures and Tables

**Table 1:** Compositions of 5 different tablet formulations tested in this study.

Tablet formulations						
Formulation	Total content (mg)	Nanoparticles (mg)	Starch (mg)	SMCC (mg)	HPMC (mg)	CaCO_3_ percentage (%)
F1	500	425	35	35	5	85
F2	470	415	25	25	5	88
F3	250	210	15	20	5	84
F4	180	150	17	11	3	83
F5	90	75	8	6	1	83
